# Use of topical versus injectable anaesthesia for ShangRing circumcisions in men and boys in Kenya: Results from a randomized controlled trial

**DOI:** 10.1371/journal.pone.0218066

**Published:** 2019-08-14

**Authors:** Quentin Awori, Philip S. Li, Richard K. Lee, Daniel Ouma, Millicent Oundo, Mukhaye Barasa, Nereah Obura, David Mwamkita, Raymond Simba, Jairus Oketch, Nixon Nyangweso, Mary Maina, Nicholas Kiswi, Michael Kirui, Betty Chirchir, Marc Goldstein, Mark A. Barone

**Affiliations:** 1 Population Council, Nairobi, Kenya; 2 Center for Male Reproductive Medicine and Surgery, Department of Urology, Weill Cornell Medical College, New York Presbyterian Hospital, New York, New York, United States of America; 3 Bon Santé Consulting, Nairobi, Kenya; 4 Homa Bay Teaching and Referral Hospital, Homa Bay, Kenya; 5 Vipingo Health Centre, Vipingo, Kenya; 6 Center for Biomedical Research, Population Council, New York, New York, United States of America; IAVI, UNITED STATES

## Abstract

**Background:**

The ShangRing is a disposable, collar clamp circumcision device pre-qualified for use in men and boys 13 years and above. It has been shown to be faster than conventional circumcision with comparable adverse event (AE) rates and high client satisfaction. Voluntary medical male circumcision (VMMC) has been shown to dramatically reduce the risk of HIV acquisition in males. However, the fear of pain during circumcision is an important barrier to uptake. Use of topical anesthesia thus presents an opportunity to address this.

**Objectives:**

We sought to evaluate the safety, effectiveness and acceptability of the use of topical anaesthesia with ShangRing circumcision of men and boys 10 years of age and above.

**Methods:**

Participants were randomised 2:1 to receive topical or injectable anaesthesia. All participants underwent no-flip ShangRing circumcision. The primary outcome measure was pain. Secondary outcomes included ease of use of topical versus injectable anaesthesia, AEs and participant satisfaction.

**Results:**

Compared to the topical group, participants in the injectable group reported significantly more pain on administration of the anesthesia and at approximately 20 minutes after the procedure. In the topical group, sufficient anaesthesia with topical cream was not achieved in 21 (9.3%) cases before the start of the procedure; in another 6 (2.6%), supplementary injectable anaesthesia was required as the circumcision was being carried out. The AE rate was significantly lower (p<0.01) in the topical (0%) vs. the injectable group (4.2%). The most common AE was pain during the post-operative period. All AEs were managed conservatively and resolved without sequeale. 96.7% of participants were satisfied with the appearance of the healed penis and 100% would recommend the ShangRing to others. All seven male circumcision providers involved in the study preferred topical to injectable anaesthesia.

**Conclusions:**

Our results demonstrate the safety, improved clinical experience, effectiveness, and acceptability of the use of topical anaesthesia in ShangRing circumcision using the no-flip technique. Topical anaesthesia effectively eliminates needlestick pain from the clients’ VMMC experience and thus has the potential to increase demand for the service.

**Trial registration:**

ClinicalTrials.gov NCT02390310.

## Introduction

Male circumcision was proven to be effective in limiting the rate of female to male transmission of HIV in three randomised control trials (RCTs) by up to 60% [1–3] and could avert an estimated 3.7 million new infections between 2016–2026[4]. Moreover, this protective effect was shown to last for at least 5 to 6 years [5,6]. It has also been shown to be a cost-effective HIV prevention intervention based on large net savings from averted HIV medical costs[7]. Based on these findings, and recommendations from the World Health Organization (WHO) and the Joint United Nations Programme on HIV/AIDS (UNAIDS), 14 sub-Saharan African countries, including Kenya, have been implementing voluntary medical male circumcision (VMMC) as part of their national HIV prevention programs [8,9]. The original goal was to provide VMMC to 80% of males aged 15–49 years in the 14 countries by 2016. As part of the continued efforts towards achievement of 80% coverage, improvements in the planning [10,11], demand creation [12], service provision [13] and surgical technique have been widely researched and adopted. Despite considerable progress, these goals were not achieved in the original timeline [14]; new goals outlined in the WHO 2016–2021 VMMC framework now aim to have 90% of males 10–29 years circumcised by 2021 [15]. This translates to 27 million males in the 14 priority countries undergoing VMMC by 2020 [15,16] meaning countries will need to increase from the current rate of approximately 2.5–3 million circumcisions annually, to 5 million [15].

This framework also highlights the use of safer, simplified, and more acceptable techniques, such as VMMC devices, and methods that increase demand and improve client satisfaction with services. The ShangRing, a single use collar clamp device, is one of two male circumcision devices which have received WHO prequalification. It is currently prequalified for use in men and boys 13 years of age and above [17]. It has been shown to be faster than conventional circumcision with comparable adverse event (AE) rates and higher client satisfaction [18,19].

Barriers to the demand for and uptake of VMMC have been well-documented. A quantitative survey of 318 males age 15–49 in Zimbabwe showed that the fear of pain during circumcision was the most frequent deterrent to uptake of VMMC [20]. This has similarly been reported in other surveys in Kenya and Malawi [21–23]. Use of topical anesthetic cream presents an opportunity to address this barrier. Topical anesthesia (TA) is used with other circumcision devices. With the other WHO recommended device, PrePex, TA is applied during placement to alleviate the pain experienced by the clients in the post-operative period [24–27]. Unicirc, another circumcision device, also uses TA instead of injectable anesthesia (IA); similar to with the ShangRing, the foreskin in resected on the day of circumcision [28].

In this study, we evaluated the safety, effectiveness and acceptability of the use of TA for ShangRing circumcision in men and boys 10 years of age and above. To the best of our knowledge, this was the first study evaluating this aspect of ShangRing circumcisions in sub-Saharan Africa. Favorable results, if implemented in VMMC programmes, could lead to an increased demand for services.

## Materials and methods

### Study design and setting

This was a two arm RCT. Participants were randomised to receive TA or IA during their ShangRing circumcision. We used age stratified block randomisation with varying block sizes in a 2:1 ratio (TA:IA). The random allocation sequence was computer generated by a researcher unaffiliated with the study. In each treatment group, we recruited 50% of the participants aged 10–15 years of age, while the other 50% were above 15 years to assess any differences in response to the intervention due to age.

The study was conducted at two sites in Kenya: Homa Bay Teaching and Referral Hospital, in Homa Bay County and the Vipingo Health Center in Kilifi County. In Homa Bay, four satellite sites were included, where outreach study activities were conducted by the staff from the Homa Bay site.

### Circumcision providers

Three male circumcision providers (MCP) performed the circumcisions at each of the two study sites. MCPs from Homa Bay had performed numerous conventional (>1000 each) and ShangRing (>400 each) circumcisions, having provided routine VMMC services and been involved in numerous other ShangRing studies since 2009 [18,19,29–31]. MCPs at the Kilifi site were experienced in conventional circumcision having performed at least 200 circumcision each and had participated in one previous ShangRing study where each performed at least 100 circumcisions.

MCPs at both sites received training in the use of topical cream for anaesthesia during ShangRing circumcision before the start of the study from a physician experienced in its use. They were trained to apply the topical anesthetic cream, first within the inner mucosal layer of the foreskin (S1A Fig) and then over the cutaneous foreskin and penile shaft on the outside covering its distal half (S1B Fig). During training, each MCP completed at least 4 ShangRing circumcisions using topical anaesthesia.

### Study participants

We recruited men and boys 10 years of age and above. Participants were enrolled from individuals seeking VMMC at the study sites. Community mobilization activities around the study sites informed the public about the availability of ShangRing circumcision. This messaging was approved by the Kenya national taskforce on VMMC. To be eligible, participants needed to be uncircumcised, in good general health and free of any active sexually transmitted infections (STIs). Written informed consent was obtained from participants 18 years and above. For participants below 18 years old, written informed consent was provided by the parents and assent was obtained from those minors able to understand what participation in the study entailed. Per Kenya national guidelines, HIV testing, counselling and referral were offered to all participants but was not required for participation in the study. Participants with known sensitivity to injectable lidocaine or topical cream, or a congenital abnormality or other condition which in the opinion of the medical staff prevented safe participation in the study were excluded.

### Procedures

Informed consent, screening and study enrollment took place on the first day. Participants or their parents were interviewed to gather baseline demographic information. Randomization of participants was performed immediately prior to the start of the circumcision. Participants assigned to the IA group underwent dorsal penile nerve block with a penile shaft ring block, using 1% lidocaine without epinephrine [32]. Up to 5 grams of a cream containing 2.5% prilocaine and 2.5% lidocaine (Global Pharmaceuticals, Impax Laboratories, Inc., Fort Washington, PA, USA) were applied to participants in the TA group (S1A and S1B Fig), who were then asked to rest as the anaesthesia took effect. During this dwell time, study staff would examine them for complete anaesthetic effect starting from approximately 20 minutes post-application and every 5–10 minutes afterwards.

Once sufficient anaesthesia was achieved, each participant underwent no-flip ShangRing circumcision [33–35]. This is a variation of the original ShangRing technique. First, the inner ring is placed inside the foreskin, around the glans penis (S1C and S1D Fig). The outer ring is then clamped around the inner ring, on the outside of the foreskin, sandwiching the foreskin between the rings (S1E Fig); there is no need to flip the foreskin over the inner ring, hence the name “no-flip.” The foreskin is then excised (S1F Fig).

Follow-up was scheduled 7 days after circumcision, at which point the ShangRing was removed, and 42 days after circumcision for a clinical exam and final exit interview.

### Outcomes

Outcome definitions and the time point at which they were assessed are shown in Table 1. The primary outcome measure was pain, as reported by participants, at various points around the time of circumcision. We used the 11-point visual analogue scale that ranged from 0 = no pain to 10 = worst pain possible.

**Table 1:** Definitions of study outcomes.

Endpoint	Definition	Assessment
Primary outcome		
Pain experienced	Highest degree of pain reported by the participants 1) during application of anaesthesia; 2) immediately before circumcision; 3) during circumcision, reported immediately after completion of the procedure, and 4) approximately 20 minutes after circumcision.	Peri–and intraoperative
Secondary outcomes		
Circumcision duration	Time taken in minutes from the insertion on the inner ring until after resection of the foreskin (exclusive of anaesthesia time)	Operative
Intraoperative difficulties	Number and nature of difficulties experienced during the circumcision and their relatedness to the anaesthesia	Operative
Adverse events (AEs)	Intraoperative and post-operative adverse events experienced that were moderate or severe	Operative and immediate postoperative
Clinical wound healing	The proportion of participants who were clinically healed at the day 42 visit (intact epithelium covering the wound as judged by the provider on visual inspection).	Day 42 postoperative
Participant satisfaction	Questions asked to the participant/parent to evaluate this included 1. What did you like about the circumcision procedure? 2. What did you NOT like about the circumcision procedure? 3. How satisfied are you with the appearance of the healed penis? 4. Would you recommend circumcision with the ShangRing to another friend/child?	Day 42 postoperative

Secondary outcomes included procedure time, ease of use of TA and IA, AEs, clinical wound healing, and participant satisfaction. We also assessed the satisfaction of circumcision providers with use of TA at the end of the study. AEs were classified based on the definitions outlined in the COSECSA/PSI/WHO AE guide to determine type and severity [36]. AEs of mild severity were considered to be within the normal range of circumcision sequelae. We modified the definition of wound dehiscence to account for the fact that no sutures are used with the ShangRing and that healing is by secondary intention [19]. Clinicians were trained and experienced in assessing the AEs from previous ShangRing studies they had been involved in.

### Statistical analysis

By planning to recruit 345 participants, we expected to have complete follow-up data on at least 300. This sample size is in line with the recommendations from the WHO for clinical evaluation of circumcision devices, which is based on an ability to rule out an AE rate of about 5% with the new use of a prequalified device [37,38]. In our case, the new use consisted of 1) utilizing topical cream for anesthesia and 2) use of the ShangRing device in boys 10–12 years of age. Prior studies that contributed data to the WHO prequalification used injectable lidocaine, and included only boys and men 13 years of age and over [18,19,30,39]. As for the younger participants (10–12 years old), we did not anticipate that the surgical procedure would be significantly affected due to the penile sizes being mostly pre-pubertal as opposed to fully developed as in the previous studies which were on older boys/men.

Our sample size was able to provide 100% power to detect a mean difference of 1 on the visual analog pain scale using a 2-sided test at the 0.05 significance level. This calculation assumes a standard deviation of 1.8, which was based on prior data [18]. For secondary endpoints, this sample size provided at least 80% power to detect absolute differences of at least 20% between the two treatment groups using two-sided tests at the 0.05 significance level. We used a significance level of 0.05 for the multiple comparisons between the two treatment groups. As per the study protocol, the analysis was performed on an as-treated basis.

One planned interim analysis was conducted by a three member Data and Safety Monitoring Board (DSMB) after 40 participants had been circumcised in each age group in the TA arm. The analysis looked at information on the progress of the study as well as data on AEs, procedure times, problems encountered during the circumcisions, and proportion of participants healed by 42 days; these were all categorized by age and treatment group. The mandate of the DSMB was to provide guidance on the continuity of the study based on findings of clear benefit or harm related to the use of the no-flip technique or TA, evidence of futility, or concerns about recruitment. After review, the DSMB recommended continuation of the study.

All analysis was done using the R statistical software, version 3.3.3 (The R Foundation for Statistical Computing, www.r-project.org/foundation/).

### Ethical approval

Ethical approval was obtained from the Weill Cornell Medical College Institutional Review Board (IRB; protocol number 1404014997) and the Kenya Medical Research Institute (KEMRI) IRB (protocol number NON-SSC 467). Regulatory approval for use of the ShangRing device was obtained from the Kenya Pharmacy and Poisons Board (protocol ref: PPB/ECCT/15/01/03/2015(29)). This study was registered on ClinicalTrials.gov, identifier: NCT02390310 before recruitment began.

## Results

### Participant flow

**Fig 1:**
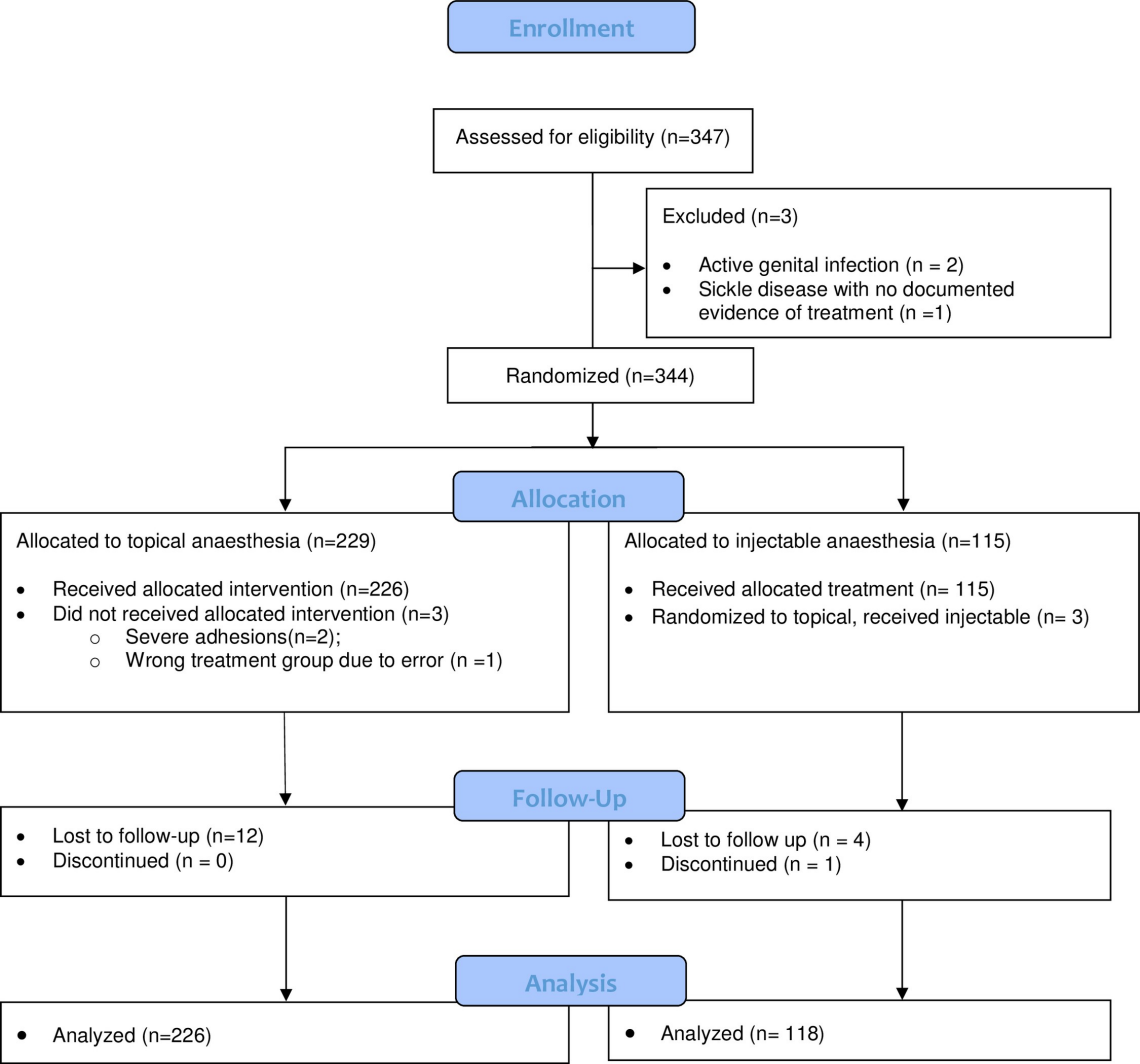
Flow of study participants.

Recruitment and follow-up took place between November 2015 and July 2016. The CONSORT diagram (Fig 1) elaborates the flow of the participants. 344/347 (99.1%) of those who volunteered and consented to participate were enrolled; three (0.9%) participants failed screening due to medical reasons. Three (0.9%) other participants, randomized to the TA group, received injectable anaesthesia; one (0.2%) due to human error and two (0.7%) due to severe penile adhesions which made adequate application of the cream inside the foreskin difficult. In all, 226 (65.1%) participants received TA and 118 (34.0%) received IA. A total of 16 participants were lost to follow-up; 12 (5.3%) in the TA and 4 (3.4%) in the IA group. One additional participant that received IA was discontinued. He experienced bleeding at the circumcision site and the cause could not be determined with the ring in place, so it was removed and the wound closed with sutures.

### Baseline characteristics

Baseline characteristics are shown in Table 2. The mean age of the study participants was 16.8±7.4 years, with the 10–12 age category alone accounting for about one third of all the participants. Almost all participants (99.7%) had attained some primary education. Personal genital hygiene (51.5%) and HIV prevention (27.6%) were the most frequently reported reasons for seeking VMMC.

**Table 2:** Baseline characteristics of participants.

Characteristic	Randomisation group		Total n (%)
	Topical n (%)	Injectable n (%)	
N	224	116	340*
Age (years)			
Mean (SD)	16.9 (7.3)	16.7 (7.5)	16.8 (7.4)
Range (Min to Max)	10–49	10–52	10–52
10–12	76 (33.9)	38 (32.8)	114 (33.5)
13–15	35 (15.6)	21 (18.1)	56 (16.5)
16–18	55 (24.6)	32 (27.6)	87 (25.6)
19–21	23 (10.3)	11 (9.5)	34 (10)
22–24	12 (5.4)	3 (2.6)	15 (4.4)
25–30	8 (3.6)	3 (2.6)	11 (3.2)
31+	15 (6.7)	8 (6.9)	23 (6.8)
Highest level of education completed			
No formal education	1(0.4)	0 (0.0)	1 (0.3)
Some Primary	113 (50.4)	63 (54.3)	176 (51.8)
Completed Primary	19 (8.5)	6 (5.2)	25 (7.4)
Some Secondary	53 (23.7)	31 (26.7)	84 (24.7)
Completed Secondary	14 (6.3)	11 (9.5)	25 (7.4)
Some College/Polytechnic	9 (4.0)	1 (0.9)	10 (2.9)
Completed College/Polytechnic	13 (5.8)	4 (3.4)	17 (5.0)
Post Graduate	2 (0.9)	0 (0.0)	2 (0.6)
Primary reason for seeking VMMC			
Hygiene	110 (49.1)	65 (56.0)	175 (51.5)
HIV Protection	64 (28.6)	30 (25.9)	94 (27.6)
Social/religious	47 (21)	20 (17.2)	67 (19.7)
Others	3 (1.2)	1 (0.9)	4 (1.2)

*Data missing from 4 participants; 2 in topical and 2 in injectable group

### Pain assessment

Pain scores reported by participants at various time points around the ShangRing circumcision are shown in Table 3. Participants in the IA group reported significantly more pain on administration of the anesthesia and at approximately 20 minutes after the procedure compared to those who received TA.

**Table 3:** Highest degree of pain experienced at various time points (0 = no pain, 10 = worst possible pain).

Time point	Topical mean (SD); N = 226	Injectable mean (SD); N = 118	p value*
Application/injection	0.02 (0.3)	1.2 (2.1)	< 0.001
Immediately before circumcision	0.1 (0.4)	0.1 (0.4)	0.51
During circumcision, reported immediately after completion of the procedure	0.2 (0.6)	0.1 (0.4)	0.14
20 minutes post-operatively	0.3 (0.7)	1.6 (1.7)	<0.001

*Student t test.

### Effectiveness of topical anaesthesia

Of the 226 participants who received TA, 199 (88.1%) circumcisions were completed successfully without any additional anaesthesia. In 21 (9.3%) cases, sufficient anaesthesia was not achieved before the start of the circumcision and supplementary IA was required. In another 6 (2.6%) cases, supplementary IA was required during circumcision. All 27 cases where TA was not successful occurred at the Kilifi study site, with 20/27 (74.1%) participants in the 10–15 age group and 7/27 (25.9%) in the over 15 group. In the IA group, 2/118 (1.7%) participants, both above 15 years of age, needed supplemental IA after the procedure had begun. We found no significant difference between the two groups in the proportion of participants who needed supplemental anaesthesia after the circumcision procedure had started (6/226 vs 2/118, Chi square test, p = 0.85). There were no allergic reactions or difficulties associated with use of the topical cream or supplemental injectable anaesthesia, when necessary.

Within the TA group, we further analysed the effectiveness of the anaesthesia by presence or absence of penile adhesions that needed to be broken down and/or phimosis requiring a dorsal slit. A significantly lower proportion of those who had penile adhesions and/or phimosis achieved sufficient anaesthesia before the start of the circumcision compared to those who did not have these conditions; 50/69 (72.5%) vs 147/155 (94.8%) respectively, Chi square test, p<0.01.

Mean±SD dwell time, the time between application of the topical cream and when ShangRing circumcision began, was 48.6±20.5 vs. 46.4±18.7 minutes in the 10–15 vs. over 15 age groups, respectively. In the IA group, it took 3.1±2.3 and 2.6±1.2 minutes for the anaesthesia to take effect in the 10–15 and over 15 age groups, respectively.

### Circumcision procedure outcomes

In both groups, all ShangRing circumcisions were completed successfully. In order to insert the inner ring, 67 (19.6%) participants needed a dorsal slit and 52 (15.3%) needed to have adhesions between the glans of the penis and inner foreskin released. There was no significant difference in the mean circumcision procedure time between the TA and IA groups (5.0 vs. 5.1 minutes respectively, Student-t test, p = 0.74). There was also no significant difference in the mean circumcision procedure time between the 10–15 and above 15 age groups; (5.0 vs. 5.1 minutes respectively, Student-t test, p = 0.61). There was only one difficulty during a circumcision, a case of severe adhesions in a participant in the 10–15 year old age group who had received IA.

### Adverse events and wound healing

There were five AEs reported during the study, four moderate and one severe (Table 4). The overall AE rate was 1.5%. The most common AE was pain during the post-operative period. The AE rate was significantly lower in the TA group compared to the IA group (0/226; 0% vs 5/118; 4.2%, Chi square test, p<0.01). All AEs were managed conservatively and resolved without sequeale.

**Table 4:** Adverse events listing.

*Participant*	Randomization group	Age group	Occurrence	Type	Severity
*VP452*	Injectable	>15	Post-operative period	Pain	Severe
*VP491*	Injectable	>15	Post-operative period	Pain	Moderate
*VP492*	Injectable	>15	Post-operative period	Pain	Moderate
*VP507*	Injectable	>15	Day 17	Infection	Moderate
*HB636*	Injectable	>15	Post-operative period	Bleeding	Moderate

Clinical wound healing was observed in 327 (95.1%) participants; one participant was discontinued and 16 were lost to follow-up before they healed. There was no significant difference in healing status at the 42 day follow-up visit between randomization groups (86.0% vs 87.6% in the TA and IA groups respectively, Chi square test, p = 0.81). Some participants (43/327; 13.1%) missed their 42-day visit, but eventually returned after 42 days and were determined to be clinically headed.

### Participant satisfaction

There were no significant differences between randomization groups in any of the measures of satisfaction, including what participants said they liked or disliked about the ShangRing circumcision and their level of satisfaction with the appearance of the healed penis (Table 5). All participants said they would recommend ShangRing circumcision to a friend/child.

**Table 5:** Responses to questions concerning participant satisfaction.

Questions asked	Topical n(%)	Injectable n(%)	p value*
n	212	110	-
1.What did you like about the circumcision procedure?**			
No stitches were needed	109 (51.4)	64 (58.2)	0.36
No injection was needed	90 (42.5)	56 (50.9)	0.31
Less pain than expected	99 (46.7)	41 (37.3)	0.29
Improved personal hygiene	75 (35.4)	48 (43.6)	0.28
The procedure was quick	62 (29.2)	51 (46.4)	0.28
Cosmetic appearance	68 (32.1)	9 (8.2)	0.20
2. What did you NOT like about the circumcision**			
There was nothing I disliked	192 (90.6)	94 (85.5)	0.82
More pain than expected	9 (4.2)	6 (5.5)	0.20
Difficult wound care	7 (3.3)	2 (1.8)	0.20
3. How satisfied are you with the appearance of the healed penis?			
Very satisfied	208 (98.1)	104 (94.5)	0.95
Somewhat satisfied	4 (1.9)	4 (3.6)	0.56
Somewhat dissatisfied	0 (0.0)	2 (1.8)	0.22
4. Would you recommend circumcision with the ShangRing to another friend/child?			
Yes	212 (100)	110 (100)	1.00

*Student t test

**multiple responses possible

### Provider preferences

Six nurses and one physician conducted the circumcisions and removals. At the end of the study, all seven reported they preferred TA to IA for ShangRing circumcision. Important merits pointed out included longer period of effective anaesthesia, lack of pain during administration of anesthesia, no need for needles, and ease of application. Challenges highlighted included the longer duration needed to achieve anaesthesia, the relative cost of topical cream, and the need for backup supplemental injectable anesthesia.

## Discussion

Our results show that ShangRing circumcision can be successfully and safely performed using the no-flip technique with TA in boys and men age 10 years and older. We found no difference in reported pain between the two groups immediately before and during circumcision, indicating that TA and IA were equally effective once anesthetic effect was achieved. Moreover, the reported pain experienced both during application of anesthetic and then approximately 20 minutes after completion of the circumcision were significantly lower in the TA group. The former is plausible given that there is no injection used, and the latter is most likely explained by the longer duration of action of topical cream compared to injectable lidocaine. Indeed, the perception of a painful injection has long been known to be a deterrent to men seeking VMMC services [20,21,40], so it is possible that use of TA could increase demand for VMMC. The overall AE rate was low; none of the AEs were related to use of TA. Three of the five AEs seen in the IA group were pain during the post-operative period, something that may be alleviated by the longer duration of action of TA.

TA provided insufficient anesthesia to begin the circumcision procedure in approximately 10% of the participants, although all were given IA and the circumcisions were completed without problems. Interestingly, all of these cases occurred at the Kilifi study site. In discussion with the providers after the completion of the study, it appears the difference may have been due to a variation in technique used for application of the anesthetic cream between the two sites. While providers at the Kilifi site applied the cream to cover the distal half of the penile shaft per training, providers at the Homa Bay site evolved the technique by covering the entire length of the penile shaft. The latter approach may have been more effective as a larger surface area for absorption of TA was available. Much as more clinical experience with TA may yield further refinements in the application technique, thus increasing effectiveness, differences between the sites highlights the importance of standardizing provider training.

We found significantly lower effectiveness of TA in participants who had penile adhesions that needed to be broken down and/or phimosis requiring a dorsal slit compared to those who had neither. This suggests that clients with adhesions or phimosis may need more attention during application to ensure that adequate cream is placed between the foreskin and the glans, to attain optimum anaesthetic uptake.

Our findings on the effectiveness of TA for ShangRing circumcision corroborate those of another new VMMC device, Unicirc [28]. With Unicirc, a mixture of lidocaine/prilocaine was applied and allowed to dwell for 30 minutes before circumcision was carried out. No participant required injectable anaesthesia, and no intraoperative complications were reported. Similarly, in our study, there were no anesthetic-related complications, although supplemental injectable anaesthesia was required in a minority of instances. Our experience is not comparable to the use of topical cream with the PrePex device given that the foreskin is not removed at the time of PrePex placement.

The mean dwell time in both age groups among those who received TA demonstrated relatively large standard deviations, for example, some participants had their circumcision as early as 20 minutes after application of the cream, while others waited over 60 minutes. It is important to keep in mind that dwell time was not an accurate reflection of the time it took for the anaesthetic to take effect, as the providers would check on the participants at intervals, and at times had to finish attending to other duties before coming back to check on the participant. Nonetheless, this increased latency required for TA should be an important consideration in facilities that plan on using it. Programmatically, space considerations are key since clients need to wait as the anaesthesia takes effect. VMMC clinics need to be aware of the average amount of time clients may need to wait.

Use of TA did not affect conduct of the circumcisions in terms of time for the actual ShangRing procedure or occurrence of intraoperative difficulties. Because the surface of the penile shaft can be oily and more difficult to grip after application of TA, excess cream needs to be wiped off the penis before the start of the procedure, thus enabling better tissue handling.

Satisfaction was high among participants. In both treatment groups, the most frequently reported reason why participants said they liked the ShangRing procedure was that no injection was needed. It is peculiar why participants in the injectable group reported this; presumably, they were speaking theoretically since they actually received an injection. Most participants reported that there was nothing they disliked about the procedure, and almost all reported they were satisfied with the appearance of the healed penis, with all saying they would recommend ShangRing circumcision to another friend/child. Similarly, the providers also reported preferring TA over IA.

The study has several limitations. It was not possible to blind the treatment administered from either the participant or the providers; this may have had an influence on the results, particularly on the reporting of pain which is highly subjective. Additionally, there were logistical limitations which did not allow us to determine the exact dwell time necessary to achieve anesthetic effect in the TA group. Finally, providers at the Homa Bay site were more experienced that those at Kilifi in ShangRing circumcision, thus potentially influencing outcomes of the study.

## Conclusion

Our results demonstrate the safety, improved clinical experience, effectiveness, and acceptability of TA for no-flip ShangRing circumcision Participants in the topical group experienced significantly less pain during the application of the anaesthesia and in the postoperative period. There were no difficulties during circumcision procedures or AEs associated with the use of topical anaesthetic cream. Although TA was not effective in inducing anaesthesia sufficiently to start the ShangRing circumcision in all participants, there were no AEs or other problems associated with the need to switch to IA when TA alone was not effective.

TA is a viable alternative for VMMC when using the no-flip ShangRing technique in men and boys 10 years and older. It effectively eliminates needlestick pain from the clients’ VMMC experience and thus has the potential to increase demand for the service. Pertinent considerations, which may vary with service delivery points and clients, should include the dwell time needed for the topical cream to take effect.

## Supporting information

S1 Fig Use of topical cream and the no-flip ShangRing technique. Topical cream was applied on the inner surface of the foreskin (A) and then on the outer surface of the foreskin on the penile shaft covering the distal half of the penis (B). The topical cream was left to take effect for approximately 20–45 minutes before the circumcision procedure was started. First, excess cream was wiped off the shaft of the penis before the inner ring was slipped into the foreskin to the level of the coronal sulcus (C, D). The outer ring was then clamped around the inner ring, sandwiching the foreskin between (E). The foreskin was then resected (F).
(PDF)

S1 File An anonymized version of the study protocol. (PDF)

S2 File CONSORT (version 2010) checklist. (PDF)

S3 File Anonymized data set containing summary tables, used to reach the conclusions drawn in the manuscript. (DOCX)

S4 File Anonymized data set containing the raw data from which analysis was done. (XLSX)
