# Supplementary figures and images for: Use of topical versus injectable anaesthesia for ShangRing circumcisions in men and boys in Kenya: Results from a randomized controlled trial
Source: PLoS One. 2019 Aug 14;14(8):e0218066. doi: 10.1371/journal.pone.0218066 (PMC6693766; doi:10.1371/journal.pone.0218066)

S1 Fig.

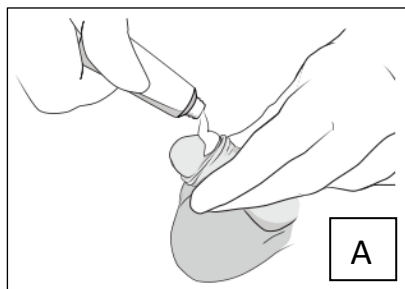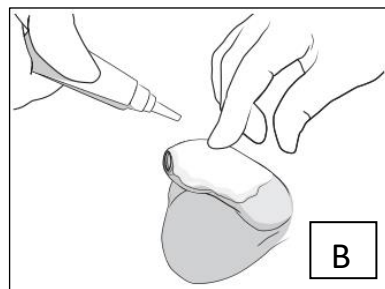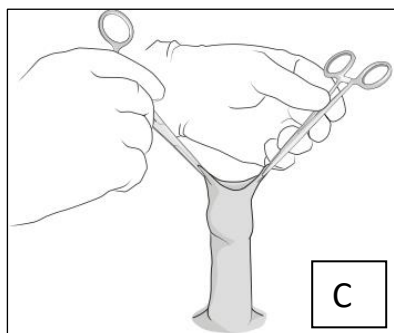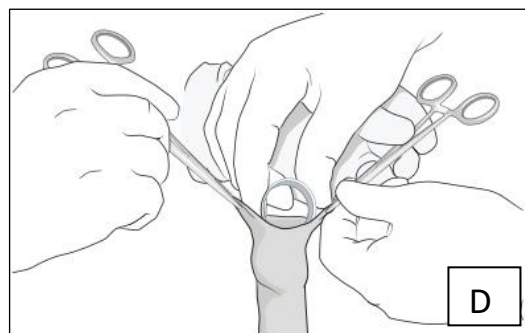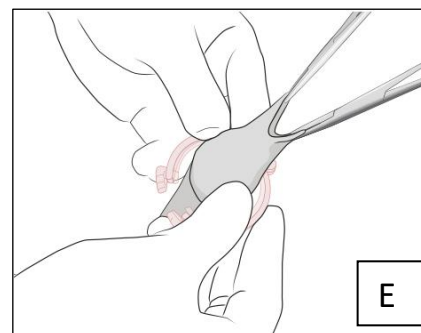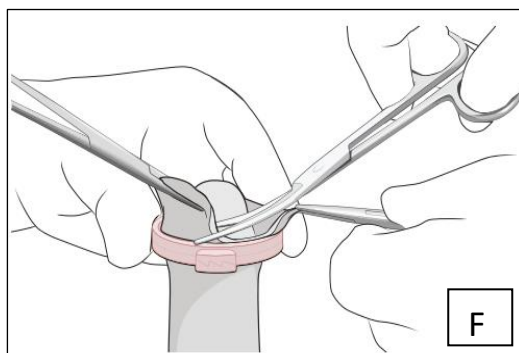

Supplement: S1 Fig — Topical cream was applied on the inner surface of the foreskin (A) and then on the outer surface of the foreskin on the penile shaft covering the distal half of the penis (B). The topical cream was left to take effect for approximately 20–45 minutes before the circumcision procedure was started. First, excess cream was wiped off the shaft of the penis before the inner ring was slipped into the foreskin to the level of the coronal sulcus (C, D). The outer ring was then clamped around the inner ring, sandwiching the foreskin between (E). The foreskin was then resected (F). (PDF) [file pone.0218066.s001.pdf]
