# Supplementary material for: Use of topical versus injectable anaesthesia for ShangRing circumcisions in men and boys in Kenya: Results from a randomized controlled trial
Source: PLoS One. 2019 Aug 14;14(8):e0218066. doi: 10.1371/journal.pone.0218066 (PMC6693766; doi:10.1371/journal.pone.0218066)
Supplement: S3 File — (DOCX) [file pone.0218066.s004.docx]

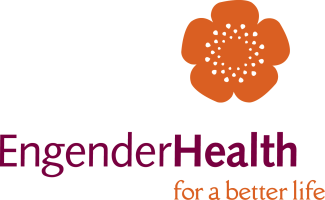

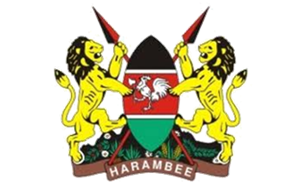

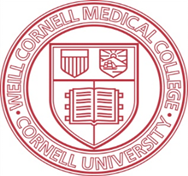

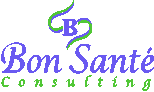


Simplifying the Shang Ring Technique for Circumcision of Men and Boys

**Phase II**

**Randomized Study of Topical vs. Injectable**

**Anesthesia for ShangRing Circumcision**

Data Tables

[redacted for submission to PLOS One]

23 June 2019

| **Sponsors:** | **EngenderHealth**  **440 Ninth Avenue**  **New York, NY 10001-1620, USA**  **Weill Cornell Medical College**  **525 E. 68th Street**  **New York, NY 10065, USA** |
| --- | --- |
| **Funded By:** | **The Bill & Melinda Gates Foundation** |

Table of Contents

[Abbreviations 4](#_Toc12309659)

[1.0 Screening, enrolment and final study status 5](#_Toc12309660)

[Table 1: Number of screened and enrolled participants by randomization group and age category 5](#_Toc12309661)

[Table 3: Number of participants enrolled by randomization group, age category and site. Intention to treat population. 5](#_Toc12309662)

[Table 4: Participant final study status by randomization group, as treated population 7](#_Toc12309663)

[Table 5: As treated population by randomization group and age category 8](#_Toc12309664)

[Table 6: Reasons for unscheduled visits by randomization group and age category. As treated population 9](#_Toc12309665)

[2.0 Demographic Information 10](#_Toc12309666)

[Table 8: Distribution of participants by randomization group and age bracket, as treated population 10](#_Toc12309667)

[Table 13: Primary reason for seeking circumcision by randomization group and age category. As treated population. 12](#_Toc12309668)

[3.0 Intake Status 13](#_Toc12309669)

[Table 21: Number of participants administered peri-op paracetamol by randomization group and age category. As treated population. 13](#_Toc12309670)

[4.0 Operation 14](#_Toc12309671)

[Table 22a: Completion of procedure under topical anaesthesia, as treated population. 14](#_Toc12309672)

[Table 23: Supplemental anaesthesia needed ONCE PROCEDURE STARTED, by randomization group, age category and site. As treated population. 15](#_Toc12309673)

[Table 24: Volume of lidocaine used (mls) in injectable group, by age category. As treated population. 16](#_Toc12309674)

[Table 25: Concentration of lidocaine used in the injectable group, by age category. As treated population. 16](#_Toc12309675)

[Table 26: Allergic reaction to anesthesia by randomization group and age category. As treated population. 16](#_Toc12309676)

[Table 27: Dwell time for anaesthetic cream in topical group, by randomization group and age category. As treated population. 17](#_Toc12309677)

[Table 28: Time for anaesthetic to take effect (minutes) in the injectable group, by randomization group and age category. As treated population. 17](#_Toc12309678)

[Table 29: Highest degree of pain experienced during application of anaesthesia, by randomization group and age category. As treated population. 18](#_Toc12309679)

[Table 30: Degree of pain experienced before start of surgery, by randomization group and age category. As treated population. 18](#_Toc12309680)

[Table 31: Duration of circumcision procedure in minutes (excludes time for anaesthesia to take effect) by randomization group and age category. As treated population. 19](#_Toc12309681)

[Table 32: Number of participants needing dorsal slit for inner ring insertion by randomization group and age category. As treated population. 19](#_Toc12309682)

[Table 33: Characterization of adhesions by randomization group and age category. As treated population. 20](#_Toc12309683)

[Table 35a: Highest degree of pain experienced DURING the procedure, by randomization group and age category. As treated population. 21](#_Toc12309684)

[5.0 Post Operation 22](#_Toc12309685)

[Table 39: Degree of post-op pain experienced, by randomization group and age category. As treated population. 22](#_Toc12309686)

[9.0 Adverse Events 23](#_Toc12309687)

[Table 59: Serious AEs, by randomization group and age category. As treated population. 23](#_Toc12309688)

[Table 60: Severity of AEs during PROCEDURE AND POST-OP PERIOD (before discharge), by randomization group and age category. As treated population. 23](#_Toc12309689)

[Table 61: Severity of adverse events during PROCEDURE AND POST-OP PERIOD, by study site and age category. As treated population. 24](#_Toc12309690)

[Table 62: Severity of adverse events during FOLLOW-UP, by randomization group and age category. As treated population. 24](#_Toc12309691)

[Table 63: Severity of adverse events during FOLLOW-UP, by study site and age category. As treated population. 24](#_Toc12309692)

[Table 64: Type of adverse events, by randomization group and age category. ALL AES IN THE STUDY. As treated population. 25](#_Toc12309693)

[Table 65: Relatedness of AEs to the ShangRing procedure, by randomization group and age category. ALL AES IN THE STUDY. As treated population. 25](#_Toc12309694)

[Table 66: Listing of AEs. All AEs in the study. 26](#_Toc12309695)

# Abbreviations

AE Adverse event

HB Homa Bay

LAR Legally authorized representative

Max Maximum

MC Male circumcision

Min Minimum

MPFU Master participant follow up

VP Vipingo, Kilifi

# Screening, enrolment and final study status

## Table 1: Number of screened and enrolled participants by randomization group and age category

| Randomization group | Topical Group | | | Injectable Group | | | Total | | |
| --- | --- | --- | --- | --- | --- | --- | --- | --- | --- |
| Age category (years) | 10-15 | > 15 | Total | 10-15 | > 15 | Total | 10-15 | > 15 | All ages |
| Number screened |  |  |  |  |  |  | 174 | 173 | 347 |
| Number enrolled | 115 | 114 | 229 | 57 | 58 | 115 | 172 | 172 | 344 |
| % of screened participants enrolled | | | | | | | 98.9% | 99.4% | 99.1% |

## Table 3: Number of participants enrolled by randomization group, age category and site. Intention to treat population.

|  | Number of participants | | | | | | | | |
| --- | --- | --- | --- | --- | --- | --- | --- | --- | --- |
| Randomization group | Topical Group* | | | Injectable Group | | | Total | | |
| Age category (years) | 10-15 | > 15 | Total | 10-15 | > 15 | Total | 10-15 | > 15 | Total |
| Study Site |  |  |  |  |  |  |  |  |  |
| Homa Bay | 77 | 78 | 155 | 39 | 43 | 82 | 116 | 121 | 237 |
| Vipingo | 38 | 36 | 74 | 18 | 15 | 33 | 56 | 51 | 107 |
| Total | 115 | 114 | 229 | 57 | 58 | 115 | 172 | 172 | 344 |

*Early in the study, two participants randomized to the topical group at Vipingo were given injectable anaesthesia. They had severe adhesions and the provider was concerned that they would not be able to get sufficient topical inside the foreskin. They decided not to attempt the procedure with the cream and instead used injectable anaesthesia. (VP414, 10-15-year-old age group and VP425, 10-15-year-old age group). In addition, one participant at Homa Bay that was randomized to the topical group was given injectable anaesthesia. It appears that this was a mistake on the part of study staff. (HB 438, 10-15-year-old age group)

**As per the protocol, the primary analyses for the study was to be conducted using an as-treated approach, i.e., depending on anesthesia method actually received, as opposed to the original random group assignment. Throughout the remaining tables, the data is presented for the “as treated” population unless otherwise indicated.**

## Table 4: Participant final study status by randomization group, as treated population

| Final study status | As treated population n (%) | | |
| --- | --- | --- | --- |
|  | Topical Group | Injectable Group | Total |
| N | 226 | 118 | 344 |
| Completed study* | 214 (94.7) | 113 (95.8) | 327 (95.1) |
| Lost to follow-up** | 12 (5.3) | 4 (3.4) | 16 (4.7) |
| Discontinued*** | 0 (0) | 1 (0.8) | 1 (0.3) |

*Completed study is defined as a participant that was determined via clinical exam to be healed at the 42 day or later visit.

** No participant was lost to follow-up before ring removal.

***2HB636 (>15-year-old age group) had bleeding approximately 30 minutes’ post-procedure (before he left the facility). The cause of the bleeding could not be ascertained with the ring in place, so the ring had to be removed and finally the wound closed with sutures. He was classified as a moderate AE, which was resolved successfully before he left the facility.

## Table 5: As treated population by randomization group and age category

| Randomization group | Topical Group N (%) | | | Injectable Group N (%) | | | Total N (%) | | |
| --- | --- | --- | --- | --- | --- | --- | --- | --- | --- |
| Age category (years) | 10-15 | > 15 | Total | 10-15 | > 15 | Total | 10-15 | > 15 | Total |
| N | 112 | 114 | 226 | 60 | 58 | 118 | 172 | 172 | 344 |
| **Final study status** | | | | | | | | | |
| Completed study | 110 (98.2) | 104 (91.2) | 214 (94.7) | 60 (100) | 53 (91.4) | 113 (95.8) | 170 (98.8) | 157 (91.3) | 327 (95.1) |
| Lost to follow-up | 2 (1.8) | 10 (8.8) | 12 (5.3) | 0 (0) | 4 (6.9) | 4 (3.4) | 2 (1.2) | 14 (8.1) | 16 (4.7) |
| Discontinued | 0 (0) | 0 (0) | 0 (0) | 0 (0) | 1 (1.7) | 1 (0.8) | 0 (0) | 1 (0.6) | 1 (0.3) |
| **Healing status at follow-up day (among the 327 who completed the study)** | | | | | | | | | |
| Healed at day 7 visit | 0 (0) | 0 (0) | 0 (0) | 0 (0) | 0 (0) | 0 (0) | 0 (0) | 0 (0) | 0 (0) |
| Healed at day 42 visit | 102 (92.7) | 82 (78.8) | 184 (86.0) | 57 (95.0) | 42 (79.2) | 99 (87.6) | 159 (93.6) | 124 (79.0) | 283 (86.5) |
| Required additional follow-up after 42 days* | 1 (0.9) | 0 (0) | 1 (0.5) | 0 (0) | 0 (0) | 0 (0) | 1 (0.6) | 0 (0) | 1 (0.3) |
| Healed at unscheduled visit after 42 days** | 7 (6.4) | 22 (21.2) | 29 (13.6) | 3 (5.0) | 11 (20.8) | 14 (12.4) | 10 (5.8) | 33 (21.0) | 43 (13.1) |
| **Number of unscheduled visits/per participant** | | | | | | | | | |
| 0 | 104 (92.9) | 92 (80.7) | 196 (86.7) | 56 (93.3) | 45 (78.9) | 102 (86.4) | 160 (93.0) | 137 (80.1) | 297 (86.6) |
| 1 | 8 (7.1) | 22 (19.3) | 30 (13.3) | 4 (6.7) | 12 (21.1) | 16 (13.6) | 12 (7.0) | 34 (19.9) | 46 (13.4) |
| Total | 112 (100) | 114 (100) | 226 (100) | 60 (100) | 57 (100) | 118 (100) | 172 (100) | 171 (100) | 343 (100)*** |

*One participant was determined not healed when he made his day 42 visit. He returned two weeks later and was healed on day 56 (HB462)

**These participants missed their 42-day visit, but eventually returned and were determined healed

***One participant was discontinued on day 0

## Table 6: Reasons for unscheduled visits by randomization group and age category. As treated population

| Randomization group | Topical Group n(%) | | | Injectable Group n(%) | | | Total n(%) | | |
| --- | --- | --- | --- | --- | --- | --- | --- | --- | --- |
| Age category (years) | 10-15 | > 15 | Total | 10-15 | > 15 | Total | 10-15 | > 15 | Total |
| Adverse event | 0 (0) | 0 (0) | 0 (0) | 1 (25.0) | 1 (8.3) | 2 (12.5) | 1 (8.3) | 1 (2.9) | 2 (4.3) |
| Delayed healing | 1 (12.5) | 0 (0) | 1 (3.3) | 0 (0) | 0 (0) | 0 (0) | 1 (8.3) | 0 (0) | 1 (2.2) |
| Missed schedule follow up visit | 7 (87.5) | 22 (100) | 29 (96.7) | 3 (75.0) | 11 (91.7) | 14 (87.5) | 10 (83.3) | 33 (97.1) | 43 (93.5) |
| **Total** | 8 (100) | 22 (100) | 30 (100) | 4 (100) | 12 (100) | 16 (100) | 12 (100) | 34 (100) | 46 (100) |

# 2.0 Demographic Information

## Table 8: Distribution of participants by randomization group and age bracket, as treated population

| Age (years) | Topical Group n (%) | Injectable Group n (%) | Total* n (%) |
| --- | --- | --- | --- |
| N | 224 | 116 | 340 |
| 10-12 | 76 (33.9) | 38 (32.8) | 114 (33.5) |
| 13-15 | 35 (15.6) | 21 (18.1) | 56 (16.5) |
| 16-18 | 55 (24.6) | 32 (27.6) | 87 (25.6) |
| 19-21 | 23 (10.3) | 11 (9.5) | 34 (10) |
| 22-24 | 12 (5.4) | 3 (2.6) | 15 (4.4) |
| 25-30 | 8 (3.6) | 3 (2.6) | 11 (3.2) |
| 31+ | 15 (6.7) | 8 (6.9) | 23 (6.8) |
| Mean (SD) | 16.9 (7.3) | 16.7 (7.5) | 16.8 (7.4) |
| Median (Interquartile Range) | 16 (12-19) | 15 (12-18) | 15.5 (12-18) |
| Range (Min to Max) | 10-49 | 10-52 | 10-52 |

*Data missing from 4 participants; 2 in topical and 2 in injectable group

## Table 13: Primary reason for seeking circumcision by randomization group and age category. As treated population.

| Randomization group | Topical Group n(%) | | | Injectable Group n(%) | | | Total n(%) | | |
| --- | --- | --- | --- | --- | --- | --- | --- | --- | --- |
| Age category (years) | 10-15 | > 15 | Total | 10-15 | > 15 | Total | 10-15 | > 15 | Total* |
| N | **110** | **114** | **224** | **58** | **58** | **116** | **168** | **172** | **340** |
| Hygiene | 60 (54.5) | 50 (43.9) | 100 (49.1) | 34 (58.6) | 31 (53.4) | 65 (56.0) | 94 (56.0) | 81 (47.1) | 175 (51.5) |
| HIV Protection | 26 (23.6) | 38 (33.3) | 64 (28.6) | 12 (20.7) | 18 (31.0) | 30 (25.9) | 38 (22.6) | 56 (32.6) | 94 (27.6) |
| Social/religious | 24 (21.8) | 23 (20.2) | 47 (21.0) | 12 (20.7) | 8 (13.8) | 20 (17.2) | 36 (21.4) | 31 (18.0) | 67 (19.7) |
| As part of medical therapy | 0 (0.0) | 1 (0.9) | 1 (0.4) | 0 (0.0) | 0 (0.0) | 0 (0.0) | 0 (0.0) | 1 (0.6) | 1 (0.3) |
| To Prevent cervical cancer | 0 (0.0) | 1 (0.9) | 1 (0.4) | 0 (0.0) | 0 (0.0) | 0 (0.0) | 0 (0.0) | 1 (0.6) | 1 (0.3) |
| To prevent cracking of the foreskin during sexual intercourse | 0 (0.0) | 1 (0.9) | 1 (0.4) | 0 (0.0) | 0 (0.0) | 0 (0.0) | 0 (0.0) | 1 (0.6) | 1 (0.3) |
| Self esteem | 0 (0.0) | 0 (0.0) | 0 (0.0) | 0 (0.0) | 1 (1.7) | 1 (0.9) | 0 (0.0) | 1 (0.6) | 1 (0.3) |

*Data missing from 4 participants; 2 in topical and 2 in injectable group

# 3.0 Intake Status

## Table 21: Number of participants administered peri-op paracetamol by randomization group and age category. As treated population.

| Randomization group | Topical Group n(%) | | | Injectable Group n(%) | | | | Total n(%) | | |
| --- | --- | --- | --- | --- | --- | --- | --- | --- | --- | --- |
| Age category (years) | 10-15 | > 15 | Total | 10-15 | > 15 | | Total | 10-15 | > 15 | Total* |
| N | **111** | **113** | **224** | **60** | **58** | | **118** | **171** | **171** | **342** |
| Pre-op | 83 (74.8%) | 83 (73.5%) | 166 (74.1%) | 44 (73.3%) | 49 (84.5%) | | 93 (78.8%) | 127 (74.3%) | 132 (77.2%) | 259 (75.7%) |
| Age category (years) | 10-15 | > 15 | Total | 10-15 | > 15 | | Total | 10-15 | > 15 | Total |
| N | **105** | **106** | **211** | **57** | **54** | | **111** | **162** | **160** | **322**** |
| Post-op | 29 (27.6%) | 34 (32.1%) | 63 (29.9%) | 19 (33.3%) | 11 (20.4%) | | 30 (27%) | 48 (29.6%) | 45 (28.1%) | 93 (28.9%) |
| If yes Dosage | | | | | | | | | | |
| 1000mg | | | |  | | 254/352 | | | | |
| 500mg | | | |  | | 98/352 | | | | |

*Data missing from 2 participants in topical group

** Data missing from 22 participants; 15 (6.6%) in the topical group and 7 (5.9%) in the injectable group

Note: It appears that 10 participants received paracetamol both before and after the procedure (5 in the topical group and 5 in the injectable group). It is unlikely that this is the case and more likely to be a data entry error. However, it could not be resolved.

# 4.0 Operation

## Table 22a: Completion of procedure under topical anaesthesia, as treated population.

| Randomization group | Topical Group | | | | | |
| --- | --- | --- | --- | --- | --- | --- |
| Age category (years) | 10-15 (n=112) | | | > 15 (n=114) | | Total (n=226)* |
|  | Homa Bay n(%) | Vipingo n(%) | Homa Bay n(%) | | Vipingo n(%) | n(%) |
| N | 76 | 36 | 78 | | 36 | 226 |
| Procedure successfully completed under topical | 76 (100) | 16 (44.4) | 78 (100) | | 29 (80.6) | 199 (88.1) |
| Procedure NOT successfully completed under topical | 0 (0) | 20 (55.6) | 0 (0) | | 7 (19.4) | 27 (11.9) |
| - Topical failed, i.e. changed to injectable before starting procedure | 0 (0) | 16 (44.4) | 0 (0) | | 5 (13.9) | 21 (9.3) |
| - Procedure started with topical, but supplemented with injectable during surgery | 0 (0) | 4 (11.1) | 0 (0) | | 2 (5.6) | 6 (2.7) |

* As this is an as-treated analysis, this table includes only those participants who were randomized to the topical group who received topical anaesthesia. It excludes the 3 participants randomized to the to the topical group who received only injectable anaesthesia.

Significantly fewer procedures were successfully completed with topical anaesthesia at Vipingo compared to at Homa Bay (p-value <0.00; X-squared = 62.069, df = 1). In addition, significantly fewer procedures were successfully completed with topical anesthesia in the younger (10-15-year-old) age group (p-value = 0.01207; X-squared = 6.3011, df = 1)

## Table 23: Supplemental anaesthesia needed ONCE PROCEDURE STARTED, by randomization group, age category and site. As treated population.

| Randomization group | Topical Group | | | Injectable Group | | | Total | | |
| --- | --- | --- | --- | --- | --- | --- | --- | --- | --- |
| Age category (years) | 10-15 | > 15 | Total | 10-15 | > 15 | Total | 10-15 | > 15 | Total* |
| N | **111 n(%)** | **113 n(%)** | **224 n(%)** | **60 n(%)** | **58 n(%)** | **118 n(%)** | **171 n(%)** | **171 n(%)** | **342 n(%)** |
| Both Sites | | | | | | | | | |
| Yes | 4 (3.6) | 2 (1.8) | 6 (2.7) ^¶^ | 0 (0) | 2 (3.4) | 2 (1.7) ^¶^ | 4 (2.3) ^±^ | 4 (2.3) ^±^ | 8 (2.3) |
| No | 107 (96.4) | 111 (98.2) | 218 (97.3) | 60 (100) | 56 (96.6) | 116 (98.3) | 167 (97.7) | 167 (97.7) | 334 (97.7) |
| Homa Bay | | | | | | | | | |
| n | 75 | 77 | 152 | 40 | 43 | 83 | 115 | 120 | 235 |
| Yes | 0 (0) | 0 (0) | 0 (0) | 0 (0) | 0 (0) | 0 (0) | 0 (0) | 0 (0) | 0 (0) |
| No | 75 (100) | 77 (100) | 152 (100) | 39 (100) | 43 (100) | 83 (100) | 115 (100) | 120 (100) | 235 (100) |
| Kilifi | | | | | | | | | |
| n | **36** | **36** | **72** | **20** | **15** | **35** | **56** | **51** | **107** |
| Yes | 4 (11.1) | 2 (5.6) | 6 (8.3) | 0 (0) | 2 (13.3) | 2 (5.7) | 4 (7.1) | 4 (7.8) | 8 (7.5) |
| No | 32 (88.9) | 34 (94.4) | 66 (91.7) | 20 (100) | 13 (86.7) | 33 (94.3) | 52 (92.9) | 47 (92.2) | 99 (92.5) |

*missing data from two participants in topical group

^¶^difference between randomization groups is not significant (p-value = 0.8447; X-squared = 0.038357, df = 1)

^±^difference between age groups is not significant (p-value = 1; X-squared = 0, df = 1)

Additional anaesthesia given during surgery

| **Site** | **Randomisation** | **Age group** | **Reason for supplementing with injectable or giving additional injectable** | **amt (mls)** |
| --- | --- | --- | --- | --- |
| VP409 | Topical | 10 to 15 | Client started feeling pain | 5 |
| VP447 | Topical | 10 to 15 | Patient started feeling pain | 6 |
| VP458 | Topical | 10 to 15 | Pain experienced during surgery once procedure had started. | 5 |
| VP463 | Topical | 10 to 15 | Pain | 6 |
| VP450 | Topical | Over 15 | Client felt pain upon incision of the dorsal slit | 10 |
| VP469 | Topical | Over 15 | Pain | 6 |
| VP491 | Injectable | Over 15 | Pain | 1 |
| VP498 | Injectable | Over 15 | Pain | 4 |

## Table 24: Volume of lidocaine used (mls) in injectable group, by age category. As treated population.

| Randomization group | Injectable Group | | |
| --- | --- | --- | --- |
| Age category (years) | 10-15 | > 15 | Total |
| N | **60** | **58** | **118** |
| Mean (mls) | 6.8* | 13.4* | 10.0 |
| SD | 2.3 | 3.5 | 4.4 |
| Min | 4 | 8 | 4 |
| Max | 14 | 25 | 25 |

*difference between age groups is significant (p-value < 0.00; t = -12.013, df = 98.092)

## Table 25: Concentration of lidocaine used in the injectable group, by age category. As treated population.

| Randomization group | Injectable Group n(%) | | |
| --- | --- | --- | --- |
| Age category (years) | 10-15 | > 15 | Total |
| N | **60** | **58** | **118** |
| One Percent | 60 | 58 | 118 |

## Table 26: Allergic reaction to anesthesia by randomization group and age category. As treated population.

| Randomization group | Topical Group n(%) | | | Injectable Group n(%) | | | Total n(%) | | |
| --- | --- | --- | --- | --- | --- | --- | --- | --- | --- |
| Age category (years) | 10-15 | > 15 | Total | 10-15 | > 15 | Total | 10-15 | > 15 | Total^*^ |
| N | **111** | **113** | **224** | **60** | **58** | **118** | **171** | **171** | **342** |
| No Reaction | 111 (100) | 113 (100) | 224 (100) | 60 (100) | 58 (100) | 118 (100) | 171 (100) | 171 (100) | 342(100) |
| Reaction | 0 (0) | 0 (0) | 0 (0) | 0 (0) | 0 (0) | 0 (0) | 0 (0) | 0 (0) | 0 (0) |

*missing data from two participants in topical group

## Table 27: Dwell time for anaesthetic cream in topical group, by randomization group and age category. As treated population.

Dwell time is the time the anaesthetic cream was on the penis before the procedure started. It was effected by multiple factors, including whether or not the participant was adequately anesthetized as well as service delivery issues such as how busy staff were at the time. It is not necessarily reflective of the time it took for the anaesthetic to take effect.

|  | Topical Group | | |
| --- | --- | --- | --- |
| Age category (years) | 10-15 | > 15 | Total* |
| **N** | **91** | **105** | **196** |
| Mean duration (in min) | 48.6 | 46.4 | 47.4 |
| SD | 20.5 | 18.7 | 19.5 |
| Min | 20 | 20 | 20 |
| Max | 146 | 103 | 146 |

*Missing data from 2 participants. Also excludes data from 7 participants due to erroneous data capture (i.e. times obviously incorrect) and data from the 21 participants where topical anaesthesia was ineffective and injectable anesthersia was administered before the procedure began.

## Table 28: Time for anaesthetic to take effect (minutes) in the injectable group, by randomization group and age category. As treated population.

|  | Injectable Group | | |
| --- | --- | --- | --- |
| Age category (years) | 10-15 | > 15 | Total* |
| N | **59** | **57** | **116** |
| Mean duration (in min) | 3.1 | 2.6 | 2.9 |
| SD | 2.3 | 1.2 | 1.9 |
| Min | 1.9 | 1 | 1 |
| Max | 16.3 | 6.4 | 16.3 |

*missing data from 2 participants

## Table 29: Highest degree of pain experienced during application of anaesthesia, by randomization group and age category. As treated population.

(0=no pain, 10=worst possible pain)

|  | | Topical Group | | | Injectable Group | | | Total | | |
| --- | --- | --- | --- | --- | --- | --- | --- | --- | --- | --- |
| Age category (years) | | 10-15 | > 15 | Total | 10-15 | > 15 | Total | 10-15 | > 15 | Total* |
| N | | **111** | **113** | **224** | **60** | **58** | **118** | 171 | 171 | 342 |
| Pain score | Mean | 0.04 | 0.01 | 0.02^¶^ | 1.4 | 1.1 | 1.2^¶^ | 0.4 | 0.4 | 0.4 |
|  | SD | 0.4 | 0.1 | 0.3 | 2.1 | 2.1 | 2.1 | 1.4 | 1.3 | 1.3 |
|  | Median | 0 | 0 | 0 | 0 | 0 | 0 | 0 | 0 | 0 |
|  | Min | 0 | 0 | 0 | 0 | 0 | 0 | 0 | 0 | 0 |
|  | Max | 4 | 1 | 4 | 7 | 7 | 7 | 7 | 7 | 7 |

*missing data from two participants in the topical group. The 21 participants in which topical anaesthesia was not effective are included here as the question about pain on administration of anaesthesia was asked immediately after the cream was applied and is unrelated to the effectiveness of the cream.

^¶^difference between randomization groups is significant (p-value < 0.00; t = -6.3167, df = 117.16)

## Table 30: Degree of pain experienced before start of surgery, by randomization group and age category. As treated population.

(0=no pain, 10=worst possible pain)

|  | | Topical Group | | | Injectable Group | | | Total | | |
| --- | --- | --- | --- | --- | --- | --- | --- | --- | --- | --- |
| Age category (years) | | 10-15 | > 15 | Total | 10-15 | > 15 | Total | 10-15 | > 15 | Total* |
| N | | **95** | **108** | **203** | **60** | **58** | **118** | **155** | **166** | **321** |
| Pain score | Mean | 0.2 | 0.0 | 0.1^¶^ | 0.1 | 0.1 | 0.1^¶^ | 0.2 | 0.0 | 0.1 |
|  | SD | 0.6 | 0.2 | 0.4 | 0.4 | 0.3 | 0.4 | 0.5 | 0.2 | 0.4 |
|  | Median | 0 | 0 | 0 | 0 | 0 | 0 | 0 | 0 | 0 |
|  | Min | 0 | 0 | 0 | 0 | 0 | 0 | 0 | 0 | 0 |
|  | Max | 4 | 1 | 4 | 2 | 2 | 2 | 4 | 2 | 4 |

*Missing data from 2 participants in the topical group. The 21 participants in which topical anaesthesia was not effective are excluded here.

^¶^difference between randomization groups is not significant (p-value = 0.5074; t = 0.66384, df = 271.19)

## Table 31: Duration of circumcision procedure in minutes (excludes time for anaesthesia to take effect) by randomization group and age category. As treated population.

|  | Topical Group | | | Injectable Group | | | Total | | |
| --- | --- | --- | --- | --- | --- | --- | --- | --- | --- |
| Age category (years) | 10-15 | > 15 | Total | 10-15 | > 15 | Total | 10-15 | > 15 | Total* |
| N | **111** | **113** | **224** | **60** | **58** | **118** | **171** | **171** | **342** |
| Mean duration (in min) | 4.9 | 5.1 | 5.0^¶^ | 5.1 | 5.1 | 5.1^¶^ | 5.0^±^ | 5.1^±^ | 5.0 |
| SD | 2.1 | 2.7 | 2.4 | 2.1 | 2.1 | 2.1 | 2.1 | 2.5 | 2.3 |
| Min | 1.5 | 2.1 | 1.5 | 2.2 | 2.0 | 2.0 | 1.5 | 2.0 | 1.5 |
| Max | 14.7 | 15.2 | 15.2 | 11.8 | 10.9 | 11.8 | 14.7 | 15.2 | 15.2 |

*missing data from two participants in the topical group

^¶^difference between randomization groups is not significant (p-value = 0.7428; t = -0.32842, df = 271.61)

^±^difference between age groups is not significant (p-value = 0.609; t = -0.51195, df = 331.88)

## Table 32: Number of participants needing dorsal slit for inner ring insertion by randomization group and age category. As treated population.

| Randomization group | Topical Group n(%) | | | Injectable Group n(%) | | | Total n(%) | | |
| --- | --- | --- | --- | --- | --- | --- | --- | --- | --- |
| Age category (years) | 10-15 | > 15 | Total | 10-15 | > 15 | Total | 10-15 | > 15 | Total* |
| N | **111** | **113** | **224** | **60** | **58** | **118** | **171** | **171** | **342** |
| Dorsal slit needed | 30 (27.1) | 11 (9.7) | 41 (18.3) ^¶^ | 20 (33.3) | 6 (10.3) | 26 (20.0) ^¶^ | 50 (29.2) ^±^ | 17 (9.9) ^±^ | 67 (19.6) |
| Mean length of slit (cm) | 1.3 | 1.5 | 1.4 | 1.4 | 1.8 | 1.5 | 1.3 | 1.6 | 1.4 |
| SD | 0.5 | 0.5 | 0.5 | 0.6 | 0.8 | 0.6 | 0.6 | 0.6 | 0.6 |
| Min | 1 | 1 | 1 | 1 | 1 | 1 | 1 | 1 | 1 |
| Max | 3 | 2 | 3 | 3 | 3 | 3 | 3 | 3 | 3 |

*missing data from two participants in the topical group

^¶^difference between randomization groups is not significant (p-value = 0.4946; X-squared = 0.46645, df = 1)

^±^difference between age groups is significant (p-value < 0.00; X-squared = 19.007, df = 1)

## Table 33: Characterization of adhesions by randomization group and age category. As treated population.

|  | Topical Group n(%) | | | Injectable Group n(%) | | | Total n(%) | | |
| --- | --- | --- | --- | --- | --- | --- | --- | --- | --- |
| Age category (years) | 10-15 | > 15 | Total | 10-15 | > 15 | Total | 10-15 | > 15 | Total |
| N | **110** | **113** | **223** | **59** | **58** | **117** | **169** | **171** | **340*** |
| Penile adhesions reported on physical exam (from intake form) | 31 (28.2) | 1 (0.9) | 32 (14.3) | 19 (32.2) | 1 (1.7) | 20 (17.1) | 50 (29.6) ^¶^ | 2 (1.2) ^¶^ | 52 (15.3) |
| Difficulty in retracting foreskin reported on physical exam (from intake form) | 16 (14.5) | 1 (0.9) | 17 (7.6) | 10 (16.9) | 4 (6.9) | 14 (12.0) | 26 (15.4) ^±^ | 5 (2.9) ^±^ | 31 (9.1) |
| N | **111** | **113** | **224** | **60** | **58** | **118** | **171** | **171** | **342**** |
| Necessary to break down adhesions (from operation form) | 47 (42.3) | 0 (0.0) | 47 (21.0) | 23 (38.3) | 1 (1.7) | 24 (20.3) | 70 (40.9) ^¥^ | 1 (0.6) ^¥^ | 71 (20.8) |
| Degree of adhesions among those where adhesions had to be broken down (from operation form) | | | | | | | | | |
| Mild | 24 (51.1) | 0 (0) | 24 (51.1) | 14 (60.9) | 0 (0) | 14 (68.3) | 38 (54.3) | 0 (0) | 38 (53.5) |
| Moderate | 20 (42.6) | 0 (0) | 20 (42.6) | 5 (21.7) | 1 (100) | 6 (24) | 25 (35.7) | 1 (100) | 26 (36.6) |
| Severe | 3 (6.4) | 0 (0) | 3 (6.4) | 4 (17.4) | 0 (0) | 4 (16.7) | 7 (10.0) | 0 (0) | 7 (9.9) |

*missing data from 4 participants; 3 in the topical and 1 in the injectable groups

**missing data from 2 participants in the topical group

^¶^difference between age groups is significant (p-value < 0.00; X-squared = 50.808, df = 1)

^±^difference between age groups is significant (p-value < 0.00; X-squared = 14.458, df = 1)

^¥^difference between age groups is significant (p-value < 0.00; X-squared = 83.333, df = 1)

## Table 35a: Highest degree of pain experienced DURING the procedure, by randomization group and age category. As treated population.

Reported immediately after completion of the procedure (0=none, 10=worst possible)

|  | | Topical Group | | | Injectable Group | | | Total | | |
| --- | --- | --- | --- | --- | --- | --- | --- | --- | --- | --- |
| Age category (years) | | 10-15 | > 15 | Total | 10-15 | > 15 | Total | 10-15 | > 15 | Total* |
| N | | **95** | **108** | **203** | **60** | **58** | **118** | **155** | **166** | **321** |
| Pain score | Mean | 0.2 | 0.1 | 0.2^¶^ | 0.1 | 0.1 | 0.1^¶^ | 0.2^±^ | 0.1^±^ | 0.2 |
|  | SD | 0.8 | 0.3 | 0.6 | 0.4 | 0.5 | 0.4 | 0.7 | 0.4 | 0.5 |
|  | Median | 0 | 0 | 0 | 0 | 0 | 0 | 0 | 0 | 0 |
|  | Min | 0 | 0 | 0 | 0 | 0 | 0 | 0 | 0 | 0 |
|  | Max | 6 | 1 | 6 | 3 | 2 | 3 | 6 | 2 | 6 |

*Missing data from 2 participants in the topical group. The 21 participants in which topical anaesthesia was not effective are excluded here.

^¶^difference between randomization groups is not significant (p-value = 0.1424; t = 1.4709, df = 299.91)

^±^difference between age groups is not significant (p-value = 0.113; t = 1.5909, df = 232.34)

# 5.0 Post Operation

## Table 39: Degree of post-op pain experienced, by randomization group and age category. As treated population.

Pain score measured approximately 20 minutes’ post-op (0=no pain, 10=worst pain possible)

|  | | Topical Group | | | Injectable Group | | | Total | | |
| --- | --- | --- | --- | --- | --- | --- | --- | --- | --- | --- |
| Age category (years) | | 10-15 | > 15 | Total | 10-15 | > 15 | Total | 10-15 | > 15 | Total* |
| N | | **88** | **100** | **188** | **57** | **54** | **111** | **145** | **154** | **299** |
| Pain score | Mean | 0.5 | 0.1 | 0.3^¶^ | 1.7 | 1.4 | 1.6^¶^ | 1.0^±^ | 0.6^±^ | 0.8 |
|  | SD | 0.9 | 0.4 | 0.7 | 1.6 | 1.9 | 1.7 | 1.3 | 1.3 | 1.3 |
|  | Median | 0 | 0 | 0 | 2 | 1 | 1 | 0 | 0 | 0 |
|  | Min | 0 | 0 | 0 | 0 | 0 | 0 | 0 | 0 | 0 |
|  | Max | 4 | 3 | 4 | 5 | 8 | 8 | 5 | 8 | 8 |

*Missing data on 24 participants; 16 (7.8%) in the topical group and 8 (6.8%) in the injectable group. The 21 participants in which topical anaesthesia was not effective are excluded here.

^¶^difference between randomization groups is significant (p-value < 0.00; t = -7.4541, df = 131.41)

^±^difference between age groups is not significant (p-value = 0.007711; t = 2.6828, df = 295.87)

# 9.0 Adverse Events

## Table 59: Serious AEs, by randomization group and age category. As treated population.

| Randomization group | Topical group n(%) | | | Injectable group n(%) | | | Total n(%) | | |
| --- | --- | --- | --- | --- | --- | --- | --- | --- | --- |
| Age category (years) | 10-15 | >15 | Total | 10-15 | > 15 | Total | 10-15 | >15 | Total |
| N | **112** | **114** | **226** | **60** | **58** | **118** | **172** | **172** | **344** |
| Serious | 0 (0) | 0 (0) | 0 (0) | 0 (0) | 0 (0) | 0 (0) | 0 (0) | 0 (0) | 0 (0) |

## Table 60: Severity of AEs during PROCEDURE AND POST-OP PERIOD (before discharge), by randomization group and age category. As treated population.

| Randomization group | Topical group n(%) | | | Injectable group n(%) | | | Total n(%) | | |
| --- | --- | --- | --- | --- | --- | --- | --- | --- | --- |
| Age category (years) | 10-15 | > 15 | Total | 10-15 | > 15 | Total | 10-15 | > 15 | Total |
| N | **112** | **114** | **226** | **60** | **58** | **118** | **172** | **172** | **344** |
| Total number of AEs | 0 (0) | 0 (0) | 0 (0) ^¶^ | 0 (0) | 4 (6.9) | 4 (3.4) ^¶^ | 0 (0) ^±^ | 4 (2.3) ^±^ | 4 (1.2) |
| Moderate | 0 (0) | 0 (0) | 0 (0) | 0 (0) | 3 (5.2) | 3 (2.5) | 0 (0) | 3 (1.7) | 3 (0.9) |
| Severe | 0 (0) | 0 (0) | 0 (0) | 0 (0) | 1 (1.7) | 1 (0.8) | 0 (0) | 1 (0.6) | 1 (0.3) |

All of these AEs were reported during the post-op period; there were no AEs reported during the ShangRing circumcision procedures

^¶^difference between randomization groups is significant (p-value = 0.02417; X-squared = 5.0822, df = 1)

^±^difference between age groups is not significant (p-value = 0.1314; X-squared = 2.2765, df = 1)

## Table 61: Severity of adverse events during PROCEDURE AND POST-OP PERIOD, by study site and age category. As treated population.

| Site | Homa Bay n(%) | | | Vipingo n(%) | | | Total n(%) | | |
| --- | --- | --- | --- | --- | --- | --- | --- | --- | --- |
| Age category (years) | 10-15 | > 15 | Total | 10-15 | > 15 | Total | 10-15 | > 15 | Total |
| N | **116** | **121** | **237** | **56** | **51** | **107** | **172** | **172** | **344** |
| Total number of AEs | 0 (0) | 1 (0.8) | 1 (0.4) ^¶^ | 0 (0) | 3 (5.9) | 3 (2.8) ^¶^ | 0 (0) | 4 (2.3) | 4 (1.2) |
| *Moderate* | 0 (0) | 1 (0.8) | 1 (0.4) | 0 (0) | 2 (3.9) | 2 (1.9) | 0 (0) | 3 (1.7) | 3 (0.9) |
| *Severe* | 0 (0) | 0 (0) | 0 (0) | 0 (0) | 1 (2.0) | 1 (0.9) | 0 (0) | 1 (0.6) | 1 (0.3) |

Any event was counted only once using highest severity. No participant had more than one adverse event.

^¶^difference between sites is not significant (p-value = 0.1725; X-squared = 1.8615, df = 1)

## Table 62: Severity of adverse events during FOLLOW-UP, by randomization group and age category. As treated population.

| Randomization group | Topical group n(%) | | | Injectable group n(%) | | | Total n (%) | | |
| --- | --- | --- | --- | --- | --- | --- | --- | --- | --- |
| Age category (years) | 10-15 | >15 | Total | 10-15 | > 15 | Total | 10-15 | >15 | Total |
| N | **112** | **114** | **226** | **60** | **58** | **118** | **172** | **172** | **344** |
| Total number of AEs | 0 (0) | 0 (0) | 0 (0) ^¶^ | 1 (1.7) | 0 (0) | 1 (0.8) ^¶^ | 1 (0.6) ^±^ | 0 (0) ^±^ | 1 (0.3) |
| *Moderate* | 0 (0) | 0 (0) | 0 (0) | 1 (1.7) | 0 (0) | 1 (0.8) | 1 (0.6) | 0 (0) | 1 (0.3) |
| *Severe* | 0 (0) | 0 (0) | 0 (0) | 0 (0) | 0 (0) | 0 (0) | 0 (0) | 0 (0) | 0 (0) |

Any event was counted only once using highest severity. No participant had more than one adverse event.

^¶^difference between randomization groups is not significant (p-value = 0.7405; X-squared = 0.10966, df = 1)

^±^difference between age groups is not significant (p-value = 1; X-squared = 0, df = 1)

## Table 63: Severity of adverse events during FOLLOW-UP, by study site and age category. As treated population.

| Site | Homa Bay n(%) | | | Vipingo n(%) | | | Total n(%) | | |
| --- | --- | --- | --- | --- | --- | --- | --- | --- | --- |
| Age category (years) | 10-15 | > 15 | Total | 10-15 | > 15 | Total | 10-15 | > 15 | Total |
| N | **116** | **121** | **237** | **56** | **51** | **107** | **172** | **172** | **344** |
| Total number of AEs | 0 (0) | 0 (0) | 0 (0) ^¶^ | 1 (1.8) | 0 (0) | 1 (0.9) ^¶^ | 1 (0.6) | 0 (0) | 1 (0.3) |
| *Moderate* | 0 (0) | 0 (0) | 0 (0) | 1 (1.8) | 0 (0) | 1 (0.9) | 1 (0.6) | 0 (0) | 1 (0.3) |
| *Severe* | 0 (0) | 0 (0) | 0 (0) | 0 (0) | 0 (0) | 0 (0) | 0 (0) | 0 (0) | 0 (0) |

Any event was counted only once using highest severity. No participant had more than one adverse event.

^¶^difference between sites is not significant (p-value = 0.6827; X-squared = 0.16709, df = 1)

## Table 64: Type of adverse events, by randomization group and age category. ALL AES IN THE STUDY. As treated population.

| Randomization group | Topical group n(%) | | | Injectable group (%) | | | Total n(%) | | |
| --- | --- | --- | --- | --- | --- | --- | --- | --- | --- |
| Age category (years) | 10-15 | > 15 | Total | 10-15 | > 15 | Total | 10-15 | > 15 | Total |
| N | **112** | **114** | **226** | **60** | **58** | **118** | **172** | **172** | **344** |
| Total number of AEs | 0 (0) | 0 (0) | 0 (0) ^¶^ | 1 (1.7) | 4 (6.9) | 5 (4.2) ^¶^ | 1 (0.6) ^±^ | 4 (2.3) ^±^ | 5 (1.5) |
| Pain | 0 (0) | 0 (0) | 0 (0) | 0 (0) | 3 (5.2) | 3 (2.5) | 0 (0) | 3 (1.7) | 3 (0.9) |
| Bleeding | 0 (0) | 0 (0) | 0 (0) | 0 (0) | 1 (1.7) | 1 (0.8) | 0 (0) | 1 (0.6) | 1 (0.3) |
| Infection | 0 (0) | 0 (0) | 0 (0) | 1 (1.7) | 0 (0) | 1 (0.8) | 1 (0.6) | 0 (0) | 1 (0.3) |

Any event was counted only once using highest severity. No participant had more than one adverse event.

^¶^difference between randomization groups is significant (p-value = 0.00822; X-squared = 6.9844, df = 1)

^±^difference between age groups is not significant (p-value = 0.3676; X-squared = 0.8118, df = 1)

## Table 65: Relatedness of AEs to the ShangRing procedure, by randomization group and age category.

| Randomization | Topical group n(%) | | | Injectable group n(%) | | | Total n(%) | | |
| --- | --- | --- | --- | --- | --- | --- | --- | --- | --- |
| Age category (years) | 10-15 | > 15 | Total | 10-15 | > 15 | Total | 10-15 | > 15 | Total |
| N | **112** | **114** | **226** | **60** | **58** | **118** | **172** | **172** | **344** |
| Definitely related | 0 (0) | 0 (0) | 0 (0) | 1 (1.7) | 4 (6.9) | 5 (4.2) | 1 (0.6) | 4 (2.3) | 5 (1.5) |

[ ALL AES IN THE STUDY. As treated population.]

## Table 66: Listing of AEs. All AEs in the study.

| # | Randomization group | Age group | Timing | Type | Severity | Serious | Related | Action taken with ShangRing | How treated | Treatment | Medication given | Indication |
| --- | --- | --- | --- | --- | --- | --- | --- | --- | --- | --- | --- | --- |
| VP452 | Injectable | >15 | Post-op | Pain | Severe | No | Definitely related | None | Clinical Management | NSAID | Diclofenac  Injection | Pain |
| VP491 | Injectable | >15 | Post-op | Pain | Moderate | No | Definitely related | None | Clinical Management | NSAID | Diclofenac  Injection | Pain |
| VP492 | Injectable | >15 | Post-op | Pain | Moderate | No | Definitely related | None | Clinical Management | NSAID | Diclofenac  injection | Pain |
| VP507 | Injectable | 10-15 | Unscheduled Day 17 | Infection | Moderate | No | Definitely related | None, device not present | Clinical management | antibiotics | Flucloxacilin | Infection |
|  |  |  |  |  |  |  |  |  |  |  | Brufen | Pain |
| HB636 | Injectable | >15 | Post-op | Bleeding | Moderate | No | Definitely related | Device removed | Clinical management | Sutured circumcision wound | None |  |

*HB636 had bleeding approximately 30 minutes’ post-procedure (before he left the facility) and because the cause of the bleeding could not be ascertained with the ring in place, the ring had to be removed and finally the wound closed with sutures. His AE was resolved successfully before he left the facility.
